# Life-course socioeconomic status and obesity: scoping review

**DOI:** 10.1186/s12963-025-00424-7

**Published:** 2025-11-18

**Authors:** Habila Adamou, Marie-Claude Paquette, Dener François, Éric Robitaille, Sékou Samadoulougou Ouindpanga, Alexandre Lebel

**Affiliations:** 1 2275 Rue de L’université, Quebec, QC G1V 0A8 Canada; 2 Center for Research in Regional Planning and Development (CRAD), Laval University, Quebec, Canada; 3 Evaluation Platform On Obesity Prevention, Quebec Heart and Lung Institute Research Center, Quebec, Canada; 4 Quebec National Institute of Public Health, Montreal, Canada; 5 Department of Nutrition, University of Montreal, Montreal, Canada; 6 Public Health Research Center, ESPUM, University of Montreal, Montreal, Canada; 7 CIUSSS du Centre-Sud-de-L ’Île-de-Montréal, Quebec, Canada; 8 CIUSSS Des Laurentides, Quebec, Canada

**Keywords:** Life-course, Socioeconomic status, Obesity, Scoping review

## Abstract

**Objectif:**

The life-course approach is believed to enhance our understanding of the intricate links between life-course socioeconomic status and obesity. In this scoping review, we delve into the literature that examines the links between life-course socioeconomic status and obesity and aim to characterize the life-course approach that was used.

**Methods:**

Our search strategy was based on the PRISMA checklist and was performed using three databases: Medline (PubMed), GeoBase (Embase), and Web of Science. We focused on studies that identify life-course socioeconomic and built environment indicators and associate them with body weight status indicators.

**Results:**

Using stringent inclusion criteria, we identified 52 relevant studies. Our analysis identified three main methodological strategies for studying the influence of life-course socioeconomic status on obesity. The main methodological approaches identified that characterize life-course approach are: 1) sensitive periods, 2) social mobility, or 3) risk accumulation. We found that low socioeconomic status in childhood, adulthood, or late adulthood; a disadvantaged socioeconomic trajectory; and cumulative exposure to socioeconomic disadvantages throughout the life-course increased the risk of obesity. Notably, the association between life-course socioeconomic status and obesity was significantly stronger for women in 56% of the studies.

**Conclusion:**

The social inequalities in obesity observed today are the outcome of socioeconomic inequalities accumulated over the life course. 56% of studies show that the influence of life-course socioeconomic status on socioeconomic inequalities in obesity is even stronger in women. Policymakers should prioritize specific interventions aimed at reducing socioeconomic disparities in obesity, particularly among women.

## Introduction

The life-course approach focuses on a sequence of events, transitions, or exposures [64]. This approach examines health as it relates to the epidemiology of peoples life history or personal trajectory. It also analyzes the impact of individual, family, or group biographies on health and social determinants [8, 71]. Life-course methodologies integrate several theories, such as social capital theory, inequality theory, and obesogenic environment theory, to study how socioeconomic and environmental factors influence health throughout the life-course. From this perspective, health is conceived as a lifelong continuum [64]. Social and historical contexts shape the development of health through socially structured pathways that make the resources that support the development of health available [26].

Obesity is a chronic disease characterized by excessive accumulation of body fat or adiposity [72]. Obesity is associated with a wide range of other chronic diseases [10, 28] and lower life expectancy [51, 68]. Social epidemiology studies consider environmental and behavioral factors to be determinants of obesity [20]. Life-course studies of obesity are based on two theoretical models: cumulative advantage/disadvantage theory and cumulative inequality theory [14]. An accumulation of social and economic disadvantage since childhood generates an accumulation of social and economic inequalities, which in turn creates inequalities in exposure to the risk of obesity. In our study, we use weight status as a generic term encompassing both body mass index, waist circumference or hip circumference (a relative measure of body shape) and adiposity (the amount and distribution of fat mass).

Obesity is measured by several indicators, such as body mass index (BMI), waist-to-hip ratio (WHR), waist circumference (WC), and left ventricular mass index. Many studies have examined the influence of environmental factors on the development of obesity [31, 47], using as unit of analysis the individual, the family, and the community (Newton et al., 2017a; [66]). The environmental factors most widely used in obesity studies are the socioeconomic status or built environments of the individual or community [16, 31], Newton et al., 2017b a;[48]). However, much of the literature is based on cross-sectional studies that include both factors [31].

Cross-sectional studies have helped to clarify the links between obesity and socioeconomic status and built environment, but they are limited in their ability to explain the persistence of obesity prevalence despite obesity prevention measures. For example, 20 out of 46 studies found no association between the food environment and obesity [9]. An emerging approach is helping to shed light on certain blind spots associated with the influence of the living environment on the occurrence of obesity [34]. The main objective of this scoping review is to explore the literature that studies the links between socioeconomic status and obesity, with a focus on studies that use a life-course approach in their methodologies.

## Methods

The six stages of our scoping review process are 1) identifying the research question; 2) identifying relevant studies; 3) selecting studies; 4) charting data; and 5) collating, summarizing, and reporting results [5, 36]. Our scoping review protocol aligns with the Preferred Reporting Items for Systematic reviews and Meta-Analyses Extension for Scoping Reviews (PRISMA-ScR) guidelines.

### Objective and eligibility criteria

This scoping review aims to explore the literature that studies the links between life-course socioeconomic status and obesity and characterize the methodological strategies used in the life-course approach. Based on [55], we aimed more specifically to.

1. Characterize the array of evidentiary sources within public health and social epidemiology domains.
2. Scrutinize the methodologies used to characterize life-course socioeconomic status.
3. Discern salient attributes or variables associated with a conceptual framework.
4. Identify and scrutinize gaps in existing knowledge in life-course socioeconomic status studies.

The study period was from January 2000 to May 2023. We searched for scientific articles written in French or English that identified the life-course socioeconomic status and built environment longitudinal indicators, then linked them with weight indicators. Table 1.

**Table 1:** Studies inclusion criteria

Concepts	Description
**Population**	All studies using human populations are included in our scoping review. Studies using simulation are excluded. Our scoping review has no restrictions on age, sex, or geographical area
**Intervention**	N/a
**Life-course indicator**	Socioeconomic life-course socioeconomics is conceptualized in three models: Socioeconomic sensitive period (childhood, early adulthood, and late adulthood), socioeconomic accumulation risk, and social mobility (socioeconomic trajectories)
**Comparaison**	We compared individual who were disadvantaged in sensitive periods, disadvantaged socioeconomic accumulation, and poor socioeconomic mobility to individual who were favorable in sensitive periods, favorable socioeconomic accumulation, and favorable socioeconomic mobility
**Outcome**	The primary outcomes of our study are all weight indicators. Life-course socioeconomic indicators and built environment longitudinal indicators
**Devis/Studies design**	At least two observation periods are necessary to trace whether they are environmental trajectories. Longitudinal or repetitive studies allow for a better understanding of the course. They can highlight certain aspects of individuals lives over the long term or repeatedly

### Search strategy

**Fig. 1:**
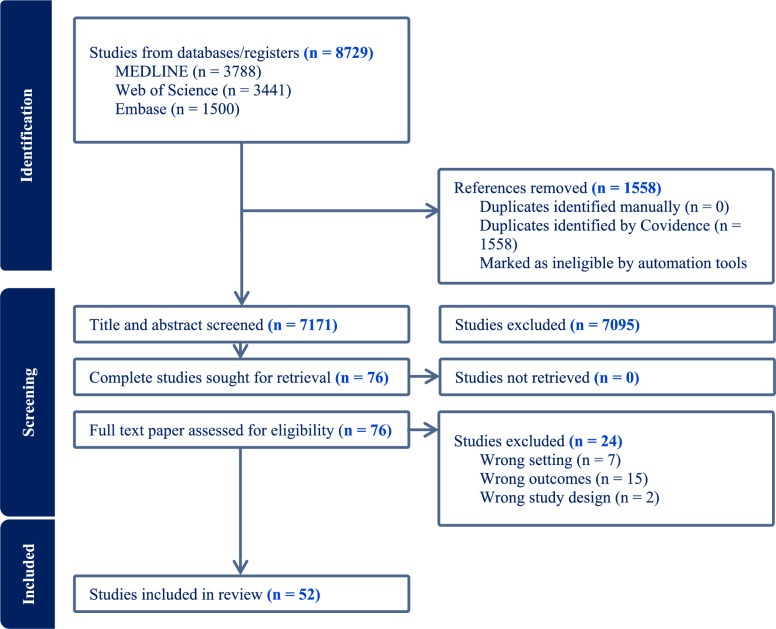
PRISMA diagram flowchart

We developed inclusion and exclusion criteria to guide the study selection process. Based on these criteria (see Table 2 for more details) [1], we used descriptors and Boolean operators to formulate search strategies in the Medline (PubMed), Web of Science, and GeoBase (Embase) databases. We used these databases because they are relevant to the topic of population health, which includes social, economic, environmental, medical, and demographic issues. Each of these databases had particularities that required a specific and adapted search strategy. The PRISMA diagram in Fig. 1 shows our article retention process using the Covidence software package by the two coauthors (HA and DF).

**Table 2:** Type of obesity indicators and methodologies used in the life-course approach

**Indicator**	**Methodological strategy**	**Number of use in studies** [56]
Life-course approach	Sensitive periods	23 (40%)
	Social mobility	12 (21%)
	Risk accumulation	10 (18%)
	Two methodologies	5 (9%)
	Three methodologies	7 (12%)

**Indicator**	**Obesity indicator**	**Number of use in studies **[60]
Obesity measures	Body mass index (BMI)	40 (66%)
	Body mass index (BMI) trajectories	5 (8%)
	Waist circumference (WC)	7 (11%)
	Hip circumference (HC)	3 (5%)
	Waist:hip ratio (WHR)	4 (6%)
	Left ventricular (LV) mass index	1 (2%)
	Gestational weight gain	1 (2%)

A study may use more than one methodological strategy and/or more than one obesity indicator. This is why the totals exceed 52, the number of studies selected

The results from the databases were exported into the Covidence software. After the results from the various databases were amalgamated, duplicates were identified and removed. Two independent reviewers (HA and DF) evaluated the database of remaining studies. An initial assessment was conducted based on the article title. Next, the reviewers determined which of those studies would move on to the abstract-reading step. From that group, each reviewer suggested a set of studies to be read in full. Finally, the reviewers reached a consensus on a list of studies to be comprehensively read; for more details, see [1].

The study selection strategy relied upon the knowledge of two independent screeners (HA and DF), with a third (AL) screener available to arbitrate and resolve any conflicts that arose during the selection process. The detailed protocol was independently reviewed and is available elsewhere [1].

### Data extraction and synthesis

Once the article retention process was completed, specific characteristics from the methods and results were extracted for each article. Below, we first provide summary statistics on the spatial and temporal distribution of the examined studies, as well as statistics on the nature of the socioeconomic indicators, the methodological strategies of the life-course approach, and the nature of the obesity indicators. Second, we discuss our main findings. We define the life-course approach and then describe the methodological strategies associated with the life-course approach used in the selected studies. We also describe the relationships between the different methodological strategies and the obesity indicators. Finally, we provide a critical analysis of the conception of sex/gender in the studies. Further details are shown in Tables 2 and 3. Table 2 identifies the studies and their construction of socioeconomic indicators, methodological strategies, obesity indicators, and analysis gender differences in obesity risk. Table 3 lists the objectives of each study, the population cohort used, observation periods and the conclusions.

**Table 3:** Methodological characteristics and main results from the selected studies

Identification	Socioeconomic & built environment indicator	Methodological strategy	Obesity indicator	Sex comparison
[56]	Childhood socioeconomic status Adult socioeconomic status Socioeconomic trajectories (upwardly mobile, persistently low, downwardly mobile, and persistently high)	Sensitive periods Social mobility	Body mass index Waist-hip ratio	No difference
[32]	Father’s social class Adult social class at 26 Adult social class at 43	Sensitive periods	Waist-hip ratio Waist-height ratio Waist circumference Body mass index	High exposure for women
[59]	Social class in childhood (parents’ education level) Social class in adulthood (adult social class)	Sensitive periods	Body mass index Waist circumference	High exposure for women
[6]	Childhood Socioeconomic position (father’s occupation) Cumulative socioeconomic position	Sensitive periods Risk accumulation	Body weight trajectories	High exposure for women
[7]	Socioeconomic trajectories (always poor, never poor, poor at birth/ non-poor at 19, and non-poor at birth/poor at 19)	Social mobility	Body mass index	High exposure for women
[24]	Socioeconomic position childhood Socioeconomic position adulthood Life-course SEP (combining information on childhood and adulthood SEP)	Sensitive periods	Body weight status	High exposure for women
[49]	Parents’ social class at 16 Own social class at 30	Sensitive periods	Overweight Obesity	High exposure for men
[57]	Childhood social class Adult social class	Sensitive periods	Body mass index	High exposure for women
[41]	Socioeconomic status trajectories (low increasing, moderate persistent, moderate increasing, and high increasing)	Social mobility	Body mass index	No difference
[18]	Childhood socioeconomic status Adulthood socioeconomic status	Sensitive periods	Body mass index Weight	High exposure for women
[13]	Childhood socioeconomic status	Sensitive periods	Weight gain over adulthood Body mass index	High exposure for women
[32]	Tertile of family income at birth Tertile of income in adulthood Family income change (1982 − 2004)	Sensitive periods	Waist circumference Hip circumference, Waist–hip ratio Body mass index	High exposure for women
[60]	Childhood socioeconomic status (parents’ occupations) Adulthood socioeconomic status (education level, occupation, household income, and marital status)	Sensitive periods	Body mass index	No difference
[63]	Using latent class analysis to define: Life-course socioeconomic status (persistent disadvantage, disadvantage with autonomy, material advantage, educational advantage, highest overall advantage)	Social mobility	Body mass index	High exposure for women
[22]	Social mobility Social accumulation	Risk accumulation Social mobility	Body mass index	High exposure for women
[45]	Childhood SEP (S1; father’s occupation when cohort member was age 4), young adult SEP (S2; cohort member’s own occupation at age 26), and later adult SEP (S3; cohort member’s own occupation at age 43)	Sensitive periods	Body mass index	High exposure for women
[62]	Social mobility groups (stable low to stable high)	Social mobility	Body mass index	High exposure for women
[19]	Socioeconomic status at 16 years Socioeconomic status at 21 years Socioeconomic status at 30 years Socioeconomic status at 43 years Cumulative socioeconomic disadvantage at each age was defined as the number of life course stages with low SES	Sensitive periods Risk accumulation	Body mass index life-course (16, 21, 30, and 43 years)	High exposure for women
[52]	Food pattern transitions Food pattern trajectory	Social mobility	Body mass index	High exposure for women
[40]	Sensitive periods (childhood SES, adulthood SES, and older SES) Accumulation of risk (cumulative socioeconomic disadvantage) Social mobility trajectory	Sensitive periods Risk accumulation Social mobility	Body mass index	High exposure for women
[17]	Total residential moves Socioeconomic change Mobility and socioeconomic interaction combined	Social mobility	Body mass index	High exposure for women
[4]	Parents’ education Young adult education Socioeconomic mobility	Sensitive periods Social mobility	Body mass index	No difference
[27]	Income longitudinal trajectories Proportion of years spent in poverty longitudinal trajectories	Social mobility	Body mass index	No difference
[33]	Family poverty status (ITNR < 2) at 2 years Family poverty status (ITNR < 2) at 15.5 years	Sensitive periods	First incidence of obesity (obesity status at 15.5 years)	No difference
[53]	Socioeconomic status at 40 years Socioeconomic status at 50 years Socioeconomic status at 60 years	Sensitive periods	Body mass index	High exposure for women
[58]	Early-life socioeconomic status	Sensitive periods	Body mass index	High exposure for women
[15]	Neighborhood disadvantage (ref: quintile 1) Annual household disposable income (ref: quintile 1) Highest educational qualification (ref: < year 12)	Sensitive periods	Body mass index trajectories	High exposure for women
[38]	Childhood socioeconomic status (father’s occupation) Young adulthood socioeconomic status (years of education) Middle/Late adulthood socioeconomic status (wealth) Cumulative advantage in socioeconomic (CAS) status Socioeconomic trajectory	Sensitive periods Risk accumulation Social mobility	Body mass index	No difference
[42]	Low obesogenicity, moderate development Moderate obesogenicity, moderate development High obesogenicity, low development	Risk accumulation	Body mass index	No difference
[3]	Life-course socioeconomic status (parents’ education and employment status)	Social mobility	Body mass index	High exposure for women
[12]	Life-course social position (upper, middle, and lower)	Social mobility	Waist circumference Body mass index	No difference
[44]	Socioeconomic position at three points during the life-course (childhood, adulthood, and late) Sensitive period models Accumulation model Social mobility models	Sensitive periods Risk accumulation Social mobility	Left ventricular mass index	No difference
[30]	Duration and timing of exposure to neighborhood disadvantage (ages 1–17, ages 1–5 (early childhood), ages 6–11 (late childhood), and ages 12–17 (adolescence))	Sensitive periods Risk accumulation	Body mass index	No difference
[54]	Accumulation: amount of time in rural, % (n) Geographic mobility (stable urban, out-migration (moving into rural area), and in-migration (moving into urban area))	Risk accumulation Social mobility	Body mass index Weight status	No difference
[65]	Long-term poverty class Neighborhood long-term poverty class	Social mobility	Body mass index	High exposure for women
[61]	Early-life socioeconomic position (childhood socioeconomic status and adulthood socioeconomic status)	Sensitive periods	Body mass index Waist circumference Waist–hip ratio	No difference
[50]	Life-course socioeconomic status (stable high, increasing, declining, and stable low)	Social mobility	Body mass index	No difference
[21]	Cumulative neighborhood deprivation Long-term mobility trajectories	Risk accumulation Social mobility	Gestational weight gain (inadequate, adequate, and excessive)	High exposure for women
[70]	Childhood socioeconomic status (education of father, education of mother) Socioeconomic trajectories (always low, decreasing, increasing, always high)	Sensitive periods Social mobility	Body mass index Waist circumference Abdominal obesity	High exposure for women
[73]	Cumulative exposure to immediate neighborhood variables (proportion non-Hispanic Black, poverty rate)	Risk accumulation	Body mass index	No difference
[29]	Neighborhood socioeconomic disadvantage in childhood (ages 6–21) and adulthood (ages 22–48) Cumulative neighborhood socioeconomic disadvantage Neighborhood socioeconomic disadvantage trajectories	Sensitive periods Risk accumulation Social mobility	Body mass index	No difference
[67]	Change in crime (less, no change, more) Change in deprivation (less, no change, more) Change in green space (less, no change, more) Change in garden (less, no change, more)	Social mobility	Continuous body mass index	No difference
[37]	Childhood poverty trajectory (stably nonpoor, stably poor, early childhood poverty with upward mobility, and early childhood nonpoor with downward mobility)	Social mobility	Body mass index	No difference by sex
[43]	Career-family sequence cluster (using parental income, parental education, etc.)	Social mobility	Body mass index	High exposure for women
[25]	Life-course of neighborhood socioeconomic status (stayed in low neighborhood socioeconomic status, fluctuated, and stayed in high neighborhood socioeconomic status)	Social mobility	Body mass index	No difference
[39]	Early social disadvantage 1934–1944 Early social disadvantage 1966 Early social disadvantage 1986	Sensitive periods	Body mass index Waist circumference	High exposure for women
[11]	Childhood socioeconomic status Early-adulthood socioeconomic status Late-adulthood socioeconomic status Accumulation of socioeconomic risk Social mobility	Sensitive periods Risk accumulation Social mobility	General obesity Abdominal obesity	No difference
[46]	Area deprivation (Townsend) (adolescence to area deprivation (Townsend) Moved between interval	Sensitive periods Social mobility	Life course body mass index (adolescence to late mid-adulthood)	No difference
[74]	Trajectory of neighborhood socioeconomic status	Social mobility	Excessive weight gain Excessive weight loss	No difference
[69]	Socioeconomic status accumulation score Socioeconomic status trajectories	Risk accumulation Social mobility	Body mass index	High exposure for women
[35]	Cumulative neighborhood deprivation Neighborhood deprivation trajectories (privileged stable, medium upward, … deprived stable)	Risk accumulation Social mobility	Obesity	High exposure for women
[23]	Latent class trajectories (low to high, consistent low, high to low, and consistent high)	Social mobility	Body mass index	No difference

## Results

### Descriptive analysis

#### Study area

The 52 articles selected are spread over five continents, but there is a marked disparity in the origin of the studies (Fig. 1). Most studies, including authors or populations, came from North America, exclusively the United States and Canada (44%, n = 23). The next most numerous studies came from authors/populations in Europe, including Sweden, Finland, and Spain (17%, n = 9) and the United Kingdom (13%, n = 7). Other studies came from Brazil (8%, n = 4), Africa (n = 2), Ghana (n = 1), and South Africa (n = 1).

#### Temporal evolution of studies addressing obesity

**Fig. 2:**
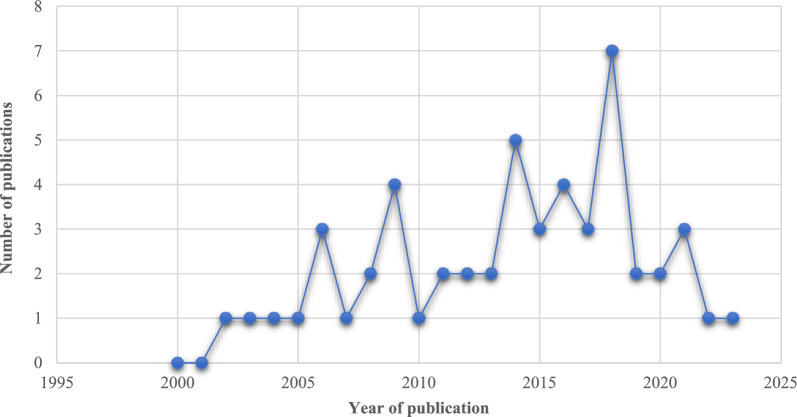
Number of studies published per year between 2000 − 2023 that analyzed life-course socioeconomic status and obesity

Within the time period, we examined, the first studies addressing obesity were published around 2002. The median number of published studies during that period was reached in 2014. From 2014 onwards, there has been an increase in publications on the topic. Almost half of all relevant studies were published between 2014 and 2021 (Fig. 2).

#### Life-course approach

“the life-course perspective addresses the balance between stability and change across the lifespan of individuals” [71]. In the studies retained for this scoping review, we have identified three methodological strategies (Table 2) that were used to study the life-course socioeconomic influence on obesity: sensitive periods (childhood, early adulthood, and late adulthood), social mobility, and risk accumulation.

The sensitive periods methodological strategy measures the effects of the three periods (childhood, early adulthood, and late adulthood) in an individuals life. For each period, the studies analyzed the influence of socioeconomic status on obesity risk (Table 2). The risk accumulation methodological strategy analyzes the duration of exposure or time spent in a particular socioeconomic status or built environment. Studies often use the duration of exposure in a disadvantaged socioeconomic status or built environment as an indicator (Table 2). The social mobility methodological strategy traces socioeconomic or built environment trajectories. Based on individuals’ socioeconomic status and built environments, this method creates clusters of life trajectories (Table 2).

### Life-course socioeconomic indicators

Among the selected studies, 49 (94%) used some socioeconomic indicators of life-course (Table 4). Of these, 16 studies used the sensitive periods methodological strategy, 15 studies used the social mobility methodological strategy, two studies used the risk accumulation methodological approach, and 16 combined the three methodological strategies (sensitive periods, social mobility, and risk accumulation). Two (4%) studies used indicators of the built environment to measure life-course socioeconomic status. including two studies used the social mobility methodological approach [42, 52]. Only one study used both life-course socioeconomic and built environment indicators [67].

**Table 4:** Data characteristics and main conclusion of selected studies

Identification	Objective	Study population	Observation periods	Conclusion
[56]	Test the hypothesis that children’s experience of socioeconomic disadvantage is associated with a wide range of health risk factors and outcomes in adult life	Cohort birth 1972–1973 in New Zealand	1972–1987	As socioeconomic status increased from childhood to adulthood, body-mass index and waist-to-hip ratio decreased
[32]	Investigate the influence of social class in childhood, young adulthood, and middle age, and intergenerational mobility on adult central and total obesity	England, Scotland, and Wales from Medical Research Council’s national survey of health and development (NSHD)	1946–1999	Intergenerational mobility indicates the potential reversibility of early life disadvantages of obesity
[59]	Investigate the association between socioeconomic position, measured at three life course stages, and obesity in the elderly	Spanish noninstitutionalized population from cross-sectional study	1941–2001	For womens educational level and adult social class with BMI and waist circumference. There was no significant association between socioeconomic circumstances throughout the life course and obesity in men
[6]	Investigate whether race differences in weight gain over 34 years were because of socioeconomic position (SEP) and psychosocial and be- behavioral factors (physical activity, cigarette smoking, alcohol consumption, depression, marital status, number of children)	Alameda County (California) Study	1965–2005	Adjustment for cumulative socioeconomic position (SEP) reduced the racial difference in weight gain among women modestly
[7]	Explored the association of height and overweight with change in socioeconomic position between birth and 19 years of age	Birth cohort in Pelotas, Brazil	1982–2001	Men who were never poor had the highest prevalence of overweight. For women, it peaked among those who were always poor and those who were never poor
[24]	Study obesity in African American women in relationship to their socioeconomic position (SEP) in childhood and adulthood	Follow-up interviews of participants in the Pitt County (North Carolina)	1988–2001	Socioeconomic position in both childhood and adulthood plays a significant role in the odds of obesity among women
[49]	Examine the cumulative influence of adverse behavioural, social, and psychosocial circumstances from adolescence to young adulthood in explaining social differences in overweight and obesity at age 30 years and if explanations differ by gender	North Sweden prospective study	14-year follow-up (beginning and end period arent mentioned)	Social inequities in overweight reflect the cumulative influence of multiple adverse circumstances experienced from adolescence to young adulthood
[57]	Assess whether childhood SEP influences disease risk in mid-life, separately from the effect of adult position, and establish how associations vary across multiple measures of disease risk	Prospective follow-up to adulthood of all born in England, Scotland and Wales	1958–2003	Child and adult social positions suggest that mortality from cardiovascular disease is influenced by social position in early life in addition to that in adult life
[41]	Examined trajectories of socioeconomic status (SES) throughout childhood and their relationship to markers of cardiovascular health in adolescence	Public high school students in the St. Louis area were recruited via school flyers	2002–2003	No association between trajectories of socioeconomic status (SES) and adolescents BMI
[18]	Examined the association between child- and adulthood socioeconomic position (SEP) and BMI and overweight/obesity in 1991 (baseline) and changes in BMI and the prevalence of overweight and obesity between 1991 and 2004	Longitudinal Dutch GLOBE study	1991–2004	Socioeconomic position (SEP) at different stages of the life course (childhood and adulthood) has an influence on body weight and weight gain in adulthood
[13]	Examine social disparities in trajectories of body mass index (BMI) over adulthood (age 18–45)	Nationally Representative Monitoring the Future Study	1986–2004	Childhood social disadvantage carries a persisting risk for increased body mass index throughout the life course, even after accounting for adult socioeconomic position
[32]	Evaluate the effects of skin colour and life-course socio-economic indicators on waist circumference (WC), hip circumference (HC) and waist:hip ratio (WHR) in young adults	Birth cohort 1982 in Pelotas (Soutern Brazil)	1982–2006	The effects of early socioeconomic position on waist circumference (WC) and circumference (HC) persist even after adjustment for adult socioeconomic position
[60]	Examine the importance of socioeconomic indicators on the development of obesity from a life course perspective	1934 and 1944 birth cohort in Helsinki, Finland	2001–2004	For men in childhood For women in adulthood
[63]	Examine racial/ethnic differences in the influence of life course SES on longitudinal obesity patterns from adolescence to adulthood	National Longitudinal Study of Adolescent Health (Add Health)	1994–2002	Females in the "persistent disadvantage" group had the highest overall risk of young adult obesity. overall risk of obesity for blacks in the "persistent disadvantage" SES group was higher compared to whites
[22]	Aims to investigate the effect of social mobility and social accumulation across three phases of the life course on prevalent overweight and obesity in adulthood	Whitehall II study	1985–1999	The odds of being overweight and obese increase with the social accumulation of disadvantage and social mobility disadvantage
[45]	To identify the life course model that best describes the association between life course socio- economic position (SEP) and cardiovascular (CVD) risk factors and explore BMI across the life course as mediators of the relationship	March 1946 birth cohort of medical Research Council NSHD	1946–1999	For women, accumulation of manual SEP across the life course was significantly associated with increasing BMI, and for men, manual SEP in childhood was associated with a significant increase in mean BMI
[62]	Aimed to examine the associations of these intergenerational SES groups with obesity, a high-risk health outcome in young adults, and compare them to traditional intergenerational SES measures	National Longitudinal Study of Adolescent Health (Add Health)	1994–2002	Growing up in a disadvantaged household and continuing that disadvantage into adulthood was associated with substantially elevated obesity risk for white females
[19]	Aims at examining whether the life course socio-economic status—body mass association in women and men is explained by the cumulative risk or adolescent sensitive period models whether associations are similar at different life course stages; and whether health behaviours explain the associations	27-year prospective cohort study	1981–2008	In women, both the cumulative risk and adolescent sensitive period models explain the socio-economic-body mass link, with associations being stronger for body mass at older age
[52]	Investigate changes in eating patterns at the individual level across three exam periods, and to prospectively examine the relation of eating trajectories to BMI at the cohort level	Framingham Heart Study cohort	1991–2001	Changes in eating patterns over time (food pattern transitions and food pattern trajectory), particularly in an unhealthful direction, are associated with higher BMI and increased risk of overweight and obesity
[40]	Elucidate the association between life-course socioeconomic status (SES) and obesity among older (aged 60 and older) Singaporean Chinese men and women	Health and Lifestyles Survey	1908–2009	Women experiencing upward social mobility have lower odds of obesity compared to those with low socioeconomic status and high socioeconomic status throughout their life-course
[17]	Determine the relationship between residential mobility and body mass index (BMI) among Black adolescents and examine the role of changes in household socioeconomic status (SES)	Birth cohort of South African urban children	1990–2005	Environmental change and increased resources to influence the risk of obesity in transitioning societies, particularly for females
[4]	Evaluated (1) whether the association between SES mobility categories and adult body mass index (BMI) varied by immigrant generation and (2) whether remaining low SES into adulthood was associated with loss of a BMI health advantage among immigrants	National Longitudinal Study of Adolescent Health (Add Health)	1994–2009	Considering SES mobility and immigrant generation is important when examining the relationship between SES and BMI
[27]	Analysed the association between poverty trajectories with body mass index (BMI) Z-scores or the risk of being overweight or obese across four ages (6 years, 8 years, 10 years and 12 years) in childhood)	Quebec Longitudinal Study of Child Development cohort	1998–2010	Childhood poverty trajectory is associated with elevated BMI Z-scores and increased odds of being overweight or obese in children
[33]	Examined the relationship between timing of poverty and risk of first-incidence obesity from ages 3 to 15.5 years	National Institute of Child Health and Human Development Study	1991–2007	Children who experienced poverty before age 2 were more likely to develop obesity later in adolescence
[53]	To test which life course model best describes the association between socioeconomic disadvantage and obesity among 60-year-old inhabitants of Västerbotten County in Northern Sweden	3340 individuals born between 1930 and 1932 was studied. Body mass index (BMI) at the age of 60 and information on socioeconomic status at three stages of life (ages 40, 50, and 60 years) was collected	1930–1990	Life-course socioeconomic disadvantaged in all stages of late adulthood is a particularly important indicator for addressing the social gradients in BMI among women in Northern Sweden
[58]	Explore reciprocal associations between socioeconomic status (SES) and body mass in the 1939 birth cohort of non-Hispanic white men and women	Wisconsin Longitudinal Study	1957–1993	Early-life socioeconomic disadvantage on midlife BMI is stronger among women compared to men
[15]	Do socioeconomic inequities in body mass index (BMI) widen across the adult life-course?	Household, Income, and Labour Dynamics in Australia	2006–2012	Males tended to have higher BMI than females on average, particularly at younger ages. Higher BMI was associated with higher levels of neighborhood socioeconomic disadvantage
[38]	This study demonstrates body mass in middle and late adulthood as a consequence of the complex interplay among individuals genes, lifetime socioeconomic experiences, and the historical context in which they live	Health and Retirement Study (HRS)	1992–2012	The findings suggest that life-course socioeconomic status influences on body mass index may depend on individuals genetic makeup
[42]	Characterized neighborhoods with respect to their composition of features, and quantified associations with diet, physical activity (PA), body mass index (BMI), and insulin resistance (IR)	Longitudinal biracial cohort	1992–2006	Neighborhood composition of food resources may be relevant for dietary behaviors and cardiometabolic outcomes— such as BMI
[3]	Examine the association between life-course socio-economic status (SES) and adult body mass index (BMI) among women in Ghana	Ghana Study of Global Ageing and Health (SAGE)	2007–2008	Higher individual and life-course SES is associated with higher BMI among women in Ghana, although maternal employment was associated with lower BMI
[12]	Aim was to investigate an individual life-course social position (LiSoP) gradient and its mediators with T2D risk in the EPIC- Spain cohort	EPIC-Spain Cohort	1992–2006	Body mass index and Waist Circumference were identified as the main mediators of the relationship between life-course social position and Type 2 diabetes mellitus risk
[44]	Investigate associations between occupational socioeconomic position during childhood, early adulthood and middle age and measures of cardiac structure [left ventricular (LV) mass index and relative wall thickness (RWT)] and function [systolic: ejection fraction (EF) and midwall fractional shortening (mFS); diastolic: left atrial (LA)volume, E/A ratio and E/e’ ratio)]	MRC National Survey of Health and Development	2006–2010	LV mass index was associated with socioeconomic position at all points across the life course. BMI may be an important mediator of the associations between socioeconomic position and cardiac structure and function
[30]	Investigates the effects of duration and timing of exposure to neighborhood disadvantage from birth through age 17 years on obesity incidence in early adulthood and black/white disparities therein	Panel Study of Income Dynamics are (PSID)	1970–2011	The duration and timing of exposure to neighborhood disadvantage during childhood and adolescence are associated with obesity incidence in early adulthood for both blacks and whites
[54]	Investigated whether body mass index (BMI) and weight status in mid-adulthood were predicted by trajectories of urban–rural residence from childhood to adulthood	Participants aged 7e15 years in 1985 (n ¼ 8498) were followed up in 2004–2006 (n ¼ 3999, aged 26–36 years) and 2009–2011 (n ¼ 3049, aged 31–41 years)	2004–2011	Trajectories of urban–rural residence from childhood to adulthood have a significant impact on body mass index (BMI) and weight status in mid-adulthood
[65]	investigate how long-term latent neighborhood poverty trajectories predict the likelihood of obesity, considering short-term individual residential mobility	Californian mothers American Community Survey	1970–2009	Those who lived in long-term low-poverty tracts or were upwardly mobile had significantly lower odds of being obese compared to those who lived in long-term high-poverty tracts
[61]	Aimed to assess the associations of childhood and adulthood socioeconomic position (SEP) and social mobility with cardiometabolic risk factors (CMRs) later in life	Jerusalem Perinatal Study (JPS)	1976–2009	Lower SEP in childhood is associated with a higher body mass index, while lower SEP in adulthood is associated with a higher waist-to-hip ratio (WHR)
[50]	Examine the association between SES over the life- course and the burden of cardio-metabolic risk factors in middle-income countries	World Health Organization (WHO) Study on Global Ageing and Adult Health (SAGE)	2007–2010	Higher life-course SES for both men and women was associated with increased odds of overweight/obesity
[21]	Understanding how long-term exposure to adverse neigh- borhood environments influences GWG. We estimated associations between average exposure to and trajectories of long-term neighborhood socioeconomic deprivation and risk of inadequate or excessive GWG	National Longitudinal Survey of Youth	1979–2012	Long-term exposure to neighborhood socioeconomic deprivation is associated with gestational weight gain (GWG) patterns over a womans life course
[70]	Estimate the association between socio-economic life course, body mass index (BMI), waist circumference (WC), and general and abdominal obesity in adults	Cohort study Florianopolis, southern Brazil	2009–2012	Women with a high socio-economic position had lower BMI, waist circumference, and abdominal obesity
[73]	Examines how black, Hispanic, and white individuals’ cumulative exposure to varying levels of neighborhood poverty and co-ethnic density from their mid-teens through mid-adulthood, as well as the levels of poverty and co-ethnic density in nearby, or “extralocal,” neighborhoods, are associated with their body mass index (BMI)	National Longitudinal Survey of Youth, 1979 Cohort	1980–2010	Cumulative exposure to neighborhood poverty and co-ethnic neighbors is a stronger positive predictor of BMI
[29]	Examine the association between neighborhood socioeconomic disadvantage and the risk factors and incidence of diabetes from childhood to middle age	The Young Finns Study	1980–2012	Cumulative neighborhood socioeconomic disadvantage is associated with increased cardiometabolic risk factors as such body mass index
[67]	Investigates the relationship between environment and child overweight/obesity	UK Millennium cohort Study	2003–2012	An association between the level of crime and amount of gardens in the child’s neighborhood and their BMI
[37]	Examines how childhood poverty dynamics shape the risk of adulthood overweight/obesity	Panel Study of Income Dynamics (PSID)	1968–2013	Childhood poverty trajectory has a significant impact on the risk of adulthood overweight/obesity
[43]	Examines what career and family life-course pathways during the transition to adulthood are related to developing obesity in young adulthood	U.S. nationally representative panel survey (NLSY97)	1997–2013	Different career-family pathways are related to different risks for developing obesity during young adulthood
[25]	Investigate whether neighborhood socioeconomic status (NSES during birth, childhood and adulthood is associated with CVD risk factors in adulthood	New England Family Study	1961–2007	Living in a socioeconomically disadvantaged neighborhood early in life and in adulthood was associated with BMI
[39]	Aimed to explore the association between early life and life-course exposure to social disadvantage and later life body mass index (BMI) accounting for genetic predisposition and maternal BMI	Helsinki Birth Cohort Study (HBCS) and Northern Finland Birth Cohorts born (NFBC)	1934–2004	High social disadvantage in early life appears to be associated with higher BMI in later life
[11]	Examined the associations of socioeconomic positions (SEPs) across life stages and their associated life-course models with both general and abdominal obesity	1077 respondents aged 50 years or above. Face-to face household interviews were conducted from 2014 to 2015 in Hong Kong	1960–2010	Socioeconomic position across the life course, cumulative socioeconomic position, and social mobility influence the risk of abdominal obesity among older adults Low SEP across life stages, especially in childhood, exerted contrasting effects with reduced risk of general obesity
[46]	Investigate life course relationships between body mass index (BMI) and area deprivation (addresses at each age linked to the closest census 1971–2011 Townsend score [TOWN], re-calculated to reflect consistent 2011 lower super output area boundaries)	NCDS and British Birth Cohorts	1971–2011	Associations between area deprivation and BMI were generally consistent across late adolescence and adulthood, even when considering prior tracking of BMI and area deprivation
[74]	Determine whether improvements in neighborhood SES are associated with reduced likelihoods of excessive weight gain and excessive weight loss and whether declines are associated with increased likelihoods of these weight outcomes	National Institutes of Health-AARP (formerly known as the American Association of Retired Persons) Diet and Health study (1995–2006)	1995–2006	For men and women, a significant linear association between neighborhood socioeconomic status change and weight gain and loss
[69]	Aims to assess the relationship between an individual’s socioeconomic status over their life-course and their body mass index (BMI)	Prospective study in Pelotas (Brazil) 1993 birth cohort	1993–2015	Accumulation of low socioeconomic status from birth to 22 years of age was associated with BMI at 22 years of age
[35]	Examine: cross-sectional neighborhood deprivation association with obesity Cumulative effect in the relationship between neighborhood deprivation and obesity Neighborhood deprivation trajectories association with obesity	TorSade cohort	1996–2016	For women, cumulative neighborhood deprivation and neighborhood deprivation trajectories are associated with high exposure to obesity
[23]	Examine the relationship between individuals’ neighborhood poverty exposure trajectories in childhood and the likelihood of obesity in emerging adulthood	Panel Study of Income Dynamics (PSID)	2005–2017	Long-term exposure to neighborhood poverty has a significant impact on the risk of adulthood obesity

### Outcome measurements

Body mass index (BMI) was the most widely used measure of body weight, with 92% (n = 48) of studies using it to characterize obesity risk. However, 12% (n = 6) combined body mass index with waist circumference or hip circumference to characterize obesity. Other weight status indicators used were gestational weight gain (n = 1), abdominal obesity (n = 1), left ventricular mass index (n = 1), excessive weight gain (n = 2), and excessive weight loss (n = 1). Notably, 10% (n = 5) of studies used body mass index to create weight status trajectories.

## Main results

### Life-course socioeconomic status and weight status

**Fig. 3:**
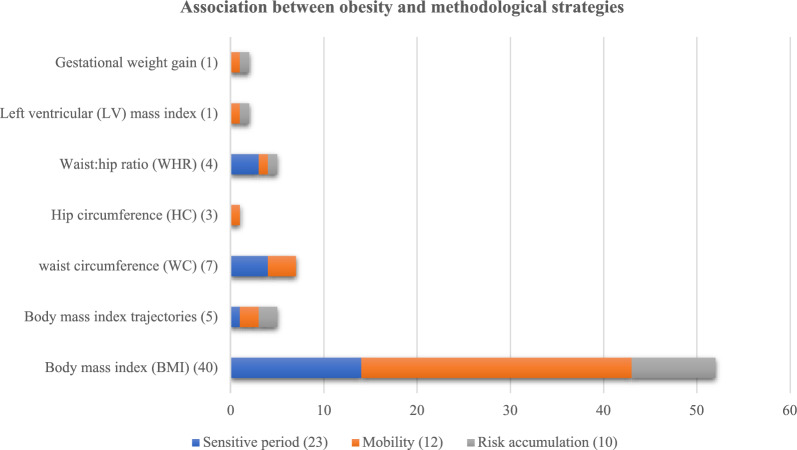
Association between obesity and methodological strategies

The selected studies used a variety of both life-course socioeconomic status indicators and weight status indicators. Nevertheless, the results in those studies overlap (Table 2 & Fig. 3). 45 studies demonstrated relationships between life-course socioeconomic status and obesity. Studies identified links between sensitive periods methodological approach and obesity, social mobility methodological approach and obesity, and risk accumulation methodological approach and obesity.

Caption: The bar chart shows the number of studies in which an association was found between the type of obesity indicators and the life-course methodological strategy used. The numbers in parentheses indicate the number of studies that reported on those methodological approaches and obesity indicators.

### Sensitive period and weight status

Studies used the occupation [6, 24, 38], E. [45, 60, 61, 70], education level [4, 13, 59, 60], household income (Gonzalez et al., 2009; [33, 49, 60]), and socioeconomic class [15, 18, 32, 39, 57, 58] of parents to measure childhood socioeconomic status [63]. For adulthood and late-adulthood socioeconomic status, the studies used occupation [24],E. [45, 60, 61], level of education [4, 38, 60], household income (Gonzalez et al., 2009; [38, 49, 60]), marital status [60], and socioeconomic class [32, 53, 57–59].

Body mass index was the main indicator used to study the relationships between sensitive periods indicators and weight status. In 23 studies, low socioeconomic status was inversely associated with body mass index in childhood [15, 32, 33],E. [45, 49, 60], adulthood [15, 32, 38],E. [45, 49, 53, 70], and late adulthood [15, 40],E. [45, 53]. Low socioeconomic status throughout the life-course was associated with high odds of obesity [15, 29, 39, 57, 58, 61]. However, all the studies showed that women with low socioeconomic status throughout their life-course have a higher likelihood of obesity. For men, associations not as constant and some studies found no association between low socioeconomic status throughout the life-course and the likelihood of obesity [24, 32, 49, 53, 59].

Some studies used waist circumference (WC) (Gonzalez et al., 2009; [39, 59, 61]), hip circumference (HC) (Gonzalez et al., 2009), and waist-to-hip ratio (WHR) [11], Gonzalez et al., 2009; [32, 61] alone or combined (Gonzalez et al., 2009; [61]) to investigate the influence of sensitive periods socioeconomic indicators on weight status. Low socioeconomic status throughout life was associated with high WC (Gonzalez et al., 2009; [59]), HC (Gonzalez et al., 2009), and WHR [11], Gonzalez et al., 2009; [32, 61]. However, women with low socioeconomic status throughout the life-course are more likely to have high WC (Gonzalez et al., 2009; [59]), HC (Gonzalez et al., 2009), and WHR (Gonzalez et al., 2009; [32]). Another study used the first incidence of obesity to determine the influence of sensitive period indicators such as weight status [33]; children who experienced poverty before two years of age were more likely to develop obesity at 15.5 years of age. Tree studies used body mass index trajectories and found that higher body mass index trajectories, such as overweight or obesity trajectories, were associated with low life-course socioeconomic status [15, 19], E. [46]. One study found that low socioeconomic status throughout life was associated with a high left ventricular (LV) mass index (E. T. [44]).

### Social mobility and weight status

Studies define social mobility in different ways, but the principle remains the same: to create similar socioeconomic groups in a dynamic perspective to trace socioeconomic or built environment trajectories. These studies include socioeconomic status trajectories [23, 38, 56, 70], neighborhood socioeconomic trajectories [25, 29, 35, 42, 65], Zhang et al., 2021), socioeconomic mobility [3, 4, 11], Ginsburg et al., 2013; [22, 27, 40, 43],E. T. [44, 54], and long-term mobility trajectories [12, 21, 50].

Most studies used body mass index to analyze the links between weight status and social mobility indicators. Socioeconomic status trajectories were inversely associated with body mass index [4, 23, 38, 56, 70]. For the same authors, a disadvantaged socioeconomic trajectory was associated with a high likelihood of obesity. Neighborhood socioeconomic trajectories showed similar results to individual socioeconomic status trajectories. Neighborhood socioeconomic trajectories were also inversely associated with body mass index [25, 29, 35, 54, 65], Zhang et al., 2021), with disadvantaged neighborhood socioeconomic trajectories associated with a high likelihood of obesity [25, 29, 35, 54, 65], Zhang et al., 2021). However, regardless of neighborhood or socioeconomic status trajectories, women from disadvantaged socioeconomic trajectories were shown to have the highest likelihood of obesity (Ginsburg et al., 2013; [35], Scharoun-Lee et al., 2009; [63, 69, 70]. Another study found there to be no association between socioeconomic status trajectories and body mass index [41].

Two studies used body mass index trajectories (E. [46, 67]). An association was found between the amount of crime, the presence of green space in a child’s neighborhood, and their continuous body mass index [67]. Long-term exposure to neighborhood socioeconomic deprivation was associated with high body mass index across late adolescence and adulthood (E. [46]). Other studies used WC to analyze the influence of social mobility on weight status [11, 12, 70]. Women who had always been in high socioeconomic positions were less likely to have abdominal obesity (based on WC), while no such association was found in men [70]. A downward socioeconomic trajectory was associated with a greater risk of abdominal obesity [11]. Another study identified WC as the main mediator of the relationship between life-course socioeconomic status and the risk of Type 2 Diabetes mellitus [12]. Other studies used other indicators of weight status. Poulton et al.[56] used WHR and found that upward mobility was associated with a low WHR. Headen et al.[21] identified a link between gestational weight gain and long-term exposure to neighborhood socioeconomic deprivation. Murray et al.(E. T. [44]) found that socioeconomic status trajectories were associated with LV mass index. Other authors found that for men and women, there was a linear association between neighborhood socioeconomic trajectories and weight gain and loss (Zhang et al., 2021).

All built environment studies used social mobility indicators: food pattern trajectory [52], neighborhood composition of food resource trajectories [42], and change in green spaces [67]. A shift towards unhealthy eating patterns over time was shown to increase the risk of being overweight and the risk of obesity [52]. Neighborhood composition of food resource trajectories may be relevant for measures of body mass index [42]. Associations were found between the continuous body mass index of children and the level of crime in their neighborhood as well as with the presence of green space in the neighborhood [67].

### Risk accumulation and weight status

The method of assessing the accumulation risk of socioeconomic status has been used extensively in recent years [11, 35],E. [46, 69]. Socioeconomic risk accumulation is assessed in studies using an indicator that captures cumulative exposure to socioeconomic and environmental characteristics. This indicator specifically reflects life-course accumulation of socioeconomic disadvantage at the individual, household, and neighborhood levels. Two studies used only the accumulation risk of socioeconomic status to analyze weight status [30, 73]. Other studies used it in combination with other methodological approaches [11, 19, 21, 22, 29, 40], E. T. [44, 69].

Some studies analyzed the cumulative exposure to disadvantages in terms of individual socioeconomic characteristics [6, 11, 19, 38, 69], while others analyzed the cumulative exposure to disadvantages in terms of neighborhood socioeconomic characteristics [21, 29, 30, 35, 73]. As in previous cases, body mass index was the most widely used indicator to analyze life-course socioeconomic status. Findings show that cumulative socioeconomic disadvantage is associated with a high body mass index [11, 19, 22, 38, 69]. Cumulative exposure to neighborhood socioeconomic deprivation is associated with a high body mass index [21, 29, 30, 35, 54]. Two studies used body mass index trajectories [6, 19]. Another found that cumulative socioeconomic advantage status led to a modest reduction in weight gain among women of different races [6], while Gustafsson et al. found that cumulative socioeconomic disadvantage status led to higher body mass index in older women[19]. Cumulative socioeconomic disadvantage was shown to influence the risk of abdominal obesity among older adults [11]. Cumulative exposure to neighborhood socioeconomic deprivation was associated with gestational weight gain (GWG) patterns over the life-course of women [21].

Among the selected studies, sex or gender was the most widely used control variable for analyzing the influence of socioeconomic life-course indicators on obesity. Prior to 2006, studies exclusively used the variable of sex to distinguish the biological dimension between male and female. The variable “gender” appeared in 2006 to distinguish the social dimension between men and women, and most studies used this variable to stratify analyses between men and women until 2015. Thereafter, the two variables (sex and gender) were used alternately in studies.

## Discussion

This scoping review aimed to explore the literature that studies the links between life-course socioeconomic status and obesity. We were able to draw two main conclusions from the selected studies.

The studies we examined mainly used three methodological strategies to measure life-course socioeconomic status: the sensitive periods, social mobility, and risk accumulation. In these studies, measures of weight status were used to analyze the influence of life-course socioeconomic status on weight status. While results showed that there are many indicators that tie these together, one clear finding emerges from all the studies: life-course socioeconomic status influences the odds of obesity. Food environment trajectories are linked to socioeconomic status [2], although few studies have used food environment indicators in the life-course approach.

Studies make extensive use of life-course sequencing, focusing on sensitive periods. In the absence of permanent longitudinal monitoring socio-economic status, the methodological solution is to subdivide the various sensitive periods of life. The sensitive periods of life make it possible to determine the successive socioeconomic status of individuals, and to estimate the impact of the successive socioeconomic status of each sensitive period on obesity. For every individual, the socioeconomic status of the neighborhood during every sensitive periods determines weight status in adulthood [39, 61]. Socioeconomic status in adulthood or late adulthood also determines weight status [39, 61]. Studies conclude that individual of low socioeconomic status in childhood and low socioeconomic status in adulthood or late adulthood have high odds of obesity. In addition, this remains to be verified over a long period between 2000 and 2020 [60, 70].

The two methodological strategy most frequently used in the selected studies are social mobility and the sensitive period. Studies analyzed social mobility by examining the socioeconomic characteristics of the neighborhood and/or the individual. Both approaches aim to group individual according to similarities in their socioeconomic trajectories. This generally leads to the creation of distributed socioeconomic trajectories: upwardly mobile, persistently low, downwardly mobile, and persistently high. This method of creating socioeconomic trajectories has been used in the same way in studies for the past 20 years [35, 56]. Most studies conclude that a disadvantaged socioeconomic trajectory is more likely to lead to obesity for an individual [25, 42, 54]. “Experiencing persistently low socioeconomic trajectory had higher odds of obesity than those with persistently high socioeconomic trajectory throughout their life-course”[37, 40].

Risk accumulation is the most recent approach and the least widely used in the studies we analyzed. This is the latest method to emerge from studies. Risk accumulation focuses on the duration and intensity of exposure to socioeconomic environments. Like the social mobility approach, the studies using the risk accumulation approach used either neighborhood or individual socioeconomic characteristics. All studies concluded that cumulative exposure to socioeconomic disadvantages throughout the life-course increases the odds of obesity [21, 69, 73].

Life-course socioeconomic status, such as sensitive periods, cumulative socioeconomic risk, and social mobility, influence the odds of obesity in late adulthood [11, 38, 65, 69]. However, the influence of life-course socioeconomic status on obesity varies according to sex or gender. Sex/gender-stratified analyses showed that women tend to be more exposed to socioeconomic inequalities throughout their life-course than men [19, 43, 53, 58]. Women of low socioeconomic status in childhood and low socioeconomic status in adulthood or late adulthood are generally at higher risk of developing obesity [18, 24],E. [45]. Women experiencing a persistently low socioeconomic trajectory had higher odds of obesity than those with a persistently high socioeconomic trajectory throughout their life-course [50, 70]. For women, cumulative exposure to socioeconomic disadvantage increases the risk of obesity [35]. Although there seemed to be some confusion between sex and gender in these studies, all of them made some distinction between men and women.

The gender variable made its appearance at the turn of 2006 in the studies selected for our scoping review. Studies turned to the use of the gender variable instead of the sex variable without the sex variable disappearing from the studies. However, this change is not followed by a conceptual shift towards a clear distinction between sex and gender. An in-depth analysis shows that all studies systematically refer to a distinction between men and women, depending on whether they use the term sex or gender in their analysis.

Another systematic review and meta-analysis by Newton et al.(Newton et al., 2017b) corroborate our findings. Based on 15 studies, the most important of which are also included in our group of selected studies, they found that women of low socioeconomic status throughout the life-course have a higher average body mass index and waist circumference than women of favorable socioeconomic status throughout the life-course (Newton et al., 2017b). This scoping review provides an updated overview of the literature on the potential impact of life-course socioeconomic status on obesity.

### Limits

The limitations to this systematic process must be considered to clearly interpret the results. Although a rigorous screening process was used to obtain the 52 studies, the limited number of databases searched, the choice of key concepts, the structure of the search equation, and the restriction to studies published in English or French are all factors that might influence our results. Because of the specific objectives of our review, some data were targeted in the data extraction process. In addition, for practical reasons, some data are not presented here, but they are available as supplementary material in the article. Future studies might include a systematic review and meta-analysis to evaluate whether the results of this study are persistent over time and space. The scoping review methodology does not require systematic assessment of study quality [5], so the absence of a systematic evaluation of study quality is another limitation. Nevertheless, the 52 selected studies can generally be considered of good quality, as reflected in their exclusive reliance on cohort data, the transparency of the statistical methods, and the clarity of the analytical variables. Despite these limitations, the conclusions of our study remain informative to understand.

## Conclusion

Exploring the literature that examines the links between life-course socioeconomic status and obesity showed that there is a high frequency of association between socioeconomic status and obesity. Low socioeconomic status in childhood and low socioeconomic status in adulthood or late adulthood increases the risk of obesity throughout the life-course. A disadvantaged socioeconomic trajectory increases the risk of obesity. Cumulative exposure to socioeconomic disadvantages throughout the life-course increases the risk of obesity.

Clearly, there is a socioeconomic inequality in the risk of obesity for disadvantaged individuals for all three life-course analysis methodological approaches. In addition, the association between life-course socioeconomic status and obesity is even stronger in women. Policymakers should prioritize specific interventions aimed at reducing socioeconomic disparities in obesity, particularly among women.

### Strengths and limitations of this study

- In this scoping review, we investigate into the literature that examines the links between life-course socioeconomic status and obesity and aim to characterize the life-course approach that were used.
- The main methodological approaches identified that characterize life-course approach are: 1) sensitive periods, 2) social mobility, or 3) risk accumulation.
- Among selected studies, 56% show that the influence of life-course socioeconomic status on socioeconomic inequalities in obesity is stronger in women.
- The sc7oping review methodology does not require systematic assessment of study quality, so the absence of a systematic evaluation of study quality is a limitation.

## Data Availability

No datasets were generated or analysed during the current study.
